# Fuzzy spherical truncation-based multi-linear protein descriptors: From their definition to application in structural-related predictions

**DOI:** 10.3389/fchem.2022.959143

**Published:** 2022-10-07

**Authors:** Ernesto Contreras-Torres, Yovani Marrero-Ponce, Julio E. Terán, Guillermin Agüero-Chapin, Agostinho Antunes, César R. García-Jacas

**Affiliations:** ^1^ Grupo de Medicina Molecular y Traslacional (MeM&T), Colegio de Ciencias de la Salud (COCSA), Escuela de Medicina, Universidad San Francisco de Quito (USFQ), Quito, Pichincha, Ecuador; ^2^ Instituto de Simulación Computacional (ISC-USFQ), Quito, Pichincha, Ecuador; ^3^ BCAM—Basque Center for Applied Mathematics, Bilbao, Spain; ^4^ Computer-Aided Molecular “Biosilico” Discovery and Bioinformatics Research International Network (CAMD-BIR IN), Quito, Ecuador; ^5^ Department of Textile Engineering, Chemistry and Science, College of Textiles, North Carolina State University, Raleigh, NC, United States; ^6^ CIIMAR—Centro Interdisciplinar de Investigação Marinha e Ambiental, Universidade do Porto, Porto, Portugal; ^7^ Departamento de Biologia, Faculdade de Ciências, Universidade do Porto, Porto, Portugal; ^8^ Cátedras Conacyt—Departamento de Ciencias de la Computación, Centro de Investigación Científica y de Educación Superior de Ensenada (CICESE), Ensenada, Baja California, Mexico

**Keywords:** 3D-protein descriptors, multi-linear algebraic forms, MuLiMs-MCoMPAs, fuzzy membership functions, fuzzy membership degree, spherical truncation, folding rate, SCOP structural classes tensor algebra-based fuzzy spherically truncated descriptors for protein Science

## Abstract

This study introduces a set of *fuzzy spherically truncated* three-dimensional (3D) multi-linear descriptors for proteins. These indices codify geometric structural information from kth *spherically truncated* spatial-(dis)similarity two-tuple and three-tuple tensors. The coefficients of these truncated tensors are calculated by applying a smoothing value to the 3D structural encoding based on the relationships between two and three amino acids of a protein embedded into a sphere. At considering, the geometrical center of the protein matches with center of the sphere, the distance between each amino acid involved in any specific interaction and the geometrical center of the protein can be computed. Then, the fuzzy membership degree of each amino acid from an spherical region of interest is computed by fuzzy membership functions (FMFs). The truncation value is finally a combination of the membership degrees from interacting amino acids, by applying the arithmetic mean as fusion rule. Several fuzzy membership functions with diverse biases on the calculation of amino acids memberships (e.g., Z-shaped (close to the center), PI-shaped (middle region), and A-Gaussian (far from the center)) were considered as well as traditional truncation functions (e.g., Switching). Such truncation functions were comparatively evaluated by exploring: 1) the frequency of membership degrees, 2) the variability and orthogonality analyses among them based on the Shannon Entropy’s and Principal Component’s methods, respectively, and 3) the prediction performance of alignment-free prediction of protein folding rates and structural classes. These analyses unraveled the singularity of the proposed fuzzy spherically truncated MDs with respect to the classical (non-truncated) ones and respect to the MDs truncated with traditional functions. They also showed an improved prediction power by attaining an external correlation coefficient of 95.82% in the folding rate modelling and an accuracy of 100% in distinguishing structural protein classes. These outcomes are better than the ones attained by existing approaches, justifying the theoretical contribution of this report. Thus, the fuzzy spherically truncated-based protein descriptors from MuLiMs-MCoMPAs (http://tomocomd.com/mulims-mcompas) are promising alignment-free predictors for modeling protein functions and properties.

## Graphical Abstract

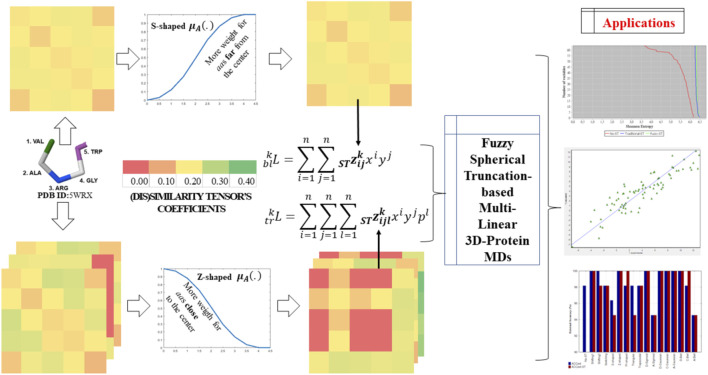

## 1 Introduction

An accurate treatment of non-bonded interatomic interactions (or relationships) is a main aspect in molecular dynamics simulations (Loncharich and Brooks, 1989; Mark and Nilsson, 2002) and to define molecular descriptors (MDs) (Todeschini et al., 2009; García-Jacas et al., 2016). Therefore, the use of functions (or rules) that truncate such interactions at a determined cutoff distance is a common practice (Sagui and Darden, 1999; Norberg and Nilsson, 2000; Patra et al., 2003; Todeschini et al., 2009). The goal of these rules is to reduce the amount of interactions, the noise in molecular encodings (García-Jacas et al., 2016), and consequently, the simulation time (Sagui and Darden, 1999). A truncation (or cutoff) rule can be applied both *abruptly* and *smoothly.* Additionally, truncation (or cutoff) rules can be applied both in a whole range (as a shifting) and in a specific interval (as a switching) (Saíz-Urra et al., 2005).

The most common truncation approach to define MDs is the application of abrupt cutoff rules (Giuliani et al., 2009; Todeschini et al., 2009; Di Paola et al., 2012). In this sense, topological/geometrical cutoffs are important in the calculation of contact order-like structural indices for proteins (Plaxco et al., 1998; Gromiha and Selvaraj, 2001; Makarov et al., 2002; Ouyang and Liang, 2008). Similarly, in the calculation of QuBiLS-MIDAS 3D descriptors (García-Jacas et al., 2020), atom-pair interactions can be truncated using rules based on topological and/or geometrical distances (García-Jacas et al., 2016). These atom-pair cutoff rules were also extended to truncate ternary and quaternary inter-atomic relationships (García-Jacas et al., 2016) for these QuBiLS-MIDAS MDs. However, the smoothed cutoff rules have brought less attention, being one of the first applications the Markov-Chain-based geometrical descriptors for proteins (Saíz-Urra et al., 2005; González-Díaz et al., 2005). These protein descriptors (PDs) truncate amino acid-pair electrostatic interactions by applying shifting- and switching-type smoothed functions (Loncharich and Brooks, 1989), denoted here as “traditional functions” (TFs).

In general, smoothing functions determine a value ranging between 0 and 1, which acts as a “truncation value” of a specific interaction. Therefore, if a truncation value tends to 1, then the analyzed interaction is closer to the lower boundary (*r*_on_) considered, whereas a truncation value with a tendency to 0, suggests the opposite (*r*_off_). Consequently, the truncation values can be deemed as defined *r*_on_-*r*_off_ intervals (membership degree). This observation encouraged the recent development of a smoothing spherical truncation method based on fuzzy membership functions (FMFs) to truncate inter-atomic relationships in small- and medium-sized molecules (García-Jacas et al., 2019). This method determines a truncation value in the interval [0, 1] by averaging the fuzzy membership degrees of interacting atoms, depending on the distance of each atom to the molecule’s geometrical center. Therefore, the truncation value weights inter atomic interactions according to the geometrical location of each atom involved depending on the chosen FMF. This geometrical location can be near to molecule’s center or far from it (molecule’s surface).

The utility of the previously-mentioned truncation scheme, implemented for the calculation of the QuBiLS-MIDAS 3D-MDs, has been shown through different chemometric studies, which have improved the modeling ability of extant 3D-MDs for small- and medium-sized molecules (García-Jacas et al., 2019). But the relevance of this truncation method has not been evaluated yet in the structural encoding of macromolecules, such as proteins.

On the other hand, the necessity of dealing with uncertainty in real wold problems has been a long-term research challenge that has originated different methods. Fuzzy Sets Theory, developed by Zadeh in 1965 (Zadeh, 1965), is a useful and appropriate approach to deal with imprecise and uncertain information in ambiguous situations. Fuzzy sets along with their extensions such as, type-2 fuzzy sets, intuitionistic fuzzy sets (IFS), intuitionistic fuzzy sets of second type (IFS2), neutrosophic fuzzy sets (NFS), Pythagorean fuzzy sets (PFS) as well as spherical fuzzy sets (SFSs, which is an advanced tool of the fuzzy sets, intuitionistic fuzzy sets and picture fuzzy sets) have provided a wide range of tools able to deal with uncertainty in different type of problems (Kutlu Gündoğdu and Kahraman, 2019; Donyatalab et al., 2020).

Our interest is focused on fuzzy sets theory (Zadeh, 1965), that allow to manage imprecise and vague information. Such vagueness is reflected by the membership degree of the objects. Fuzzy sets theory presents limitations to deal with imprecise and vague information when different sources of vagueness appear simultaneously (Donyatalab et al., 2020). Due to this fact and in order to overcome such limitations, different extensions of fuzzy sets have been introduced, which allow to simultaneously consider many types of the (non-)membership degrees. That is, despite the previous extensions overcome in different ways the managing of simultaneous sources of vagueness, all the extensions of ordinary fuzzy sets with three-dimensional membership functions that aim at defining the judgments of decision makers/experts with a more detailed description (more informatively and explicitly) are not necessary/applicable for the present problem. Here, ordinary fuzzy set (the FMFs considered were selected from the MATLAB fuzzy logic toolbox) will be use because we need only one membership grades in the truncation of interaction of amino acid for the definition of new 3D-PDs.

Geometrical 3D-PDs based on two-linear and three-linear algebraic forms were recently proposed (termed MuLiMs-MCoMPAs’ PDs) (Terán et al., 2019; Marrero-Ponce et al., 2020). To date (Contreras-Torres et al., 2019; Terán et al., 2019; Marrero-Ponce et al., 2020), these 3D-PDs are the only ones that use several (dis)similarity metrics and multi-metrics to codify chemical information from relationships among two and three amino acids (*aas*), respectively; as well as different aggregation operators to obtain global 3D-PDs from amino acid-level PDs (Terán et al., 2019; Marrero-Ponce et al., 2020). Thus, the goal of this study is to analyze the feasibility of applying FMFs in the truncation of inter-amino acid relationships for the definition of new 3D-PDs. The addition of this operation would incorporate novel and orthogonal information compared to the previous 3D-PDs for a variety of current and new applications on the protein science calculation field.

## 2 Mathematical definition of the traditional (non-truncated) geometrical multi-linear protein descriptors

The non-truncated MuLiMs-MCoMPAs 3D-PDs are computed from a protein’s conformation by codifying relationships among two (*N* = 2) and three (*N* = 3) amino acids (*aa*s) via (dis)similarity coefficients. This allows the calculation of *N*-linear (two- and three-linear) algebraic maps in ${\mathbb{R}}^{\text{N}}$ , on a canonical basis. These algebraic maps are first computed for each amino acid (*aa*) in a protein according to the following mathematical definition (see also SI1-Section 4 in Contreras-Torres et al. (2019)):

L(G)aa,kD=bl(G)aa,k(x¯,y¯)=ℤij(G)aa,kDxiyj (1)

L(G)aa,kT=tr(G)aa,k(x¯,y¯,p¯)=ℤijl(G)aa,kTxiyjpl (2)

where, *i*, *j* and *l* constitute the entries of the ℤ matrices and the $\bar{p}$ , $\bar{x}$ and $\bar{y}$ property vectors. These property vectors are comprised of the *p* ^*1*^ *,…,p* ^*n*^ *, x* ^*1*^ *,…,x* ^*n*^ and *y* ^*1*^ *,…,y* ^*n*^ coefficients (*n* is the total number of amino acids in a protein) whose values can be calculated from 16 different *aa*-level properties, including both physicochemical and folding properties, such as electronic charge (Collantes and Dunn, 1995), molecular volume (Zamyatnin, 1972), Hopp-Woods hydropathy scale (Hopp and Woods, 1981), among others (see also SI1-Section 2 in Contreras-Torres et al. (2019)) for more details). Moreover, the ℤ(G)aa,kD and ℤ(G)aa,kT terms represent the *k*th total [or group-based – (G)] two-tuple (D) and three-tuple (T) spatial (dis)similarity matrices (tensors) that are calculated for each *aa* in a protein*,* respectively (Terán et al., 2019; Marrero-Ponce et al., 2020). These *aa*-level matrices are calculated from the ℤij(G)kD and ℤ(G)kT total matrices as explained elsewhere (Terán et al., 2019; Marrero-Ponce et al., 2020). The *k* superscript denotes the exponent to which the two-tuple and three-tuple total matrices for the application of the Hadamard product. The values of *k* represent different non-covalent interactions, for instance, for *k* = −1, *k* = −2, the matrices reflect Gravitational- and Coulombic-like interactions, respectively (see SI1-Section 3.2 in Contreras-Torres et al. (2019)). The ℤ(G)kD and ℤ(G)kT total matrices of order *k* (k>1) are determined from the ℤ(G)1D and ℤ(G)1T total matrices of order 1, which hold the chemical information codified for the geometrical relationships between two and three *aas*, respectively (Terán et al., 2019; Marrero-Ponce et al., 2020). That chemical information between *aa*s is calculated via several metrics (e.g., Minkowski-type, Tanimoto, etc) for two-tuple tensors [ℤ(G)1D] , as well as via several multi-metrics (e.g., Bond Angle, Area, etc) for three-tuple tensors [ℤ(G)1T] (see SI1-Section 3.1 in Contreras-Torres et al. (2019)).

From the ℤ(G)aa,kD and ℤ(G)aa,kT *aa*-level matrices, the corresponding two-tuple (L(G)aa,kD) and three-tuple (L(G)aa,kT) *aa*-level descriptors (hereafter named as Local Amino acid Invariants, LAIs) are calculated from Eqs 1, 2, respectively, for each *aa* in a protein, and they constitute the entries of the two-tuple [L¯(G)D] and three-tuple [L¯(G)T] LAI vectors. From these LAI vectors, the global *k*th total (or group-based) two-tuple and three-tuple MuLiMs-MCoMPAs 3D-PDs can be obtained by applying different aggregation operators (e.g., geometric mean, skewness, etc) on the components of L¯(G)D and L¯(G)T , respectively. The concept of aggregation operators is based on the assumption that the best definition of a system is not necessarily additive (see SI1-Section 7 in Contreras-Torres et al. (2019)). Thus, diversity of global *k*th total (or group-based) two-linear and three-linear MuLiMs-MCoMPAs PDs can be obtained from a same LAI vector by using those mathematical operators. The calculation of the MuLiMs-MCoMPAs 3D-PDs can be performed through the MuLiMs-MCoMPAs software, which is freely available at http://tomocomd.com/mulims-mcompas (Contreras-Torres et al., 2019).

## 3 Mathematical definition of the truncated geometrical multi-linear protein descriptors based on fuzzy membership functions

Let *A* be a fuzzy set on a universe of discourse *U* (A⊆U) . This set is characterized by a fuzzy membership function, $\mu_{A}\left(a\right)$ , which associates to each element (a∈A) a real value in the interval [0, 1]. This real value represents the “membership degree” of *a* in *A* (Zadeh, 1965). If *A* is a classical set, then $\mu_{A}\left(a\right)$ is a classic characteristic function $\left[\mu_{A}\left(a\right)=1_{A}\right]$ , that is, it takes a value equal to 1 [ $\mu_{A}\left(a\right)=1$ (membership)] or equal to 0 [ $\mu_{A}\left(a\right)=0$ (non-membership)] when *a* does belong or does not belong to the *A* set, respectively (Zadeh, 1965). So, fuzzy membership functions (FMFs) are generalizations of the classic characteristic function (Zadeh, 1965), so that $\mu_{A}\left(a\right)=1$ indicates full membership, $\mu_{A}\left(a\right)=0$ indicates non-membership, and $\mu_{A}\left(a\right)\to \left(0,1\right)$ indicates partial membership that can be interpreted according to its nearness to 0 or 1.

Therefore, let *r* be the value of a relationship between *aa*s of a protein, then the mathematical definition of the fuzzy membership function-based spherical truncation method $\left[S_{N}^{fuzzy}\left(r\right)\right]$ for the MuLiMs-MCoMPAs 3D-PDs is as shown below:

$$S_{N}^{fuzzy}\left(r\right)=r\times F\left(W\right)$$ (3)

$$w_{po}=\mu_{A}\left(d_{po}\right)$$ (4)

where, *N* is the set of interacting *aa*s (*i.e.*, *aa*s involved into an inter-*aa* relationship), *r* is the value calculated for a relationship between two (r=zij(G)1D ∀zij(G)1D∈z(G)1D and i,j∈N) or among three (r=zijl(G)1T ∀zijl(G)1T∈z(G)1T and i,j,l∈N) *aa*s; W is a vector whose coefficients $\left(w_{po}\right)$ represent the fuzzy membership degree corresponding to the distance $\left(d_{po}\right)$ between each *aa* [(p=i,j,l)∈N] involved in a relationship and the geometric center (o) of the protein under study; and F(W) is the truncation value calculated via a fusion rule ***F*** (e.g., minimum, maximum, and arithmetic mean) on the W vector. As it can be noted, each coefficient $w_{po}\in W$ is calculated from an FMF, denoted as $\mu_{A}$ . It is important to remark that the arithmetic mean was the fusion (***F***) rule used in all the studies performed in this report.

Henceforth, *A* is a fuzzy set on the universe of discourse *U*, so that *U* is defined on the interval [0, *R*], where *R* is the spherical radius of each protein under study. The spherical radius (*R*) is defined as the Euclidean distance between the outermost *aa* and the geometrical center of a protein. $A=\left[d_{on},d_{off}\right]|d_{on}\geq 0$ , $d_{off}\leq R$ and $d_{on}<d_{off}$ , being $d_{on}$ and $d_{off}$ the lower and upper boundaries, respectively, of the distance-to-center interval to be considered during truncation. In this way, $\mu_{A}\left(d_{po}\right)\overset{yields}{\to }\left[0,1\right]|d_{po}\in U$ is a FMF for the *A* fuzzy set. Because different proteins will have different radiuses (*R*), then the $d_{on}$ and $d_{off}$ parameters will be calculated from percent values regarding *R*, hereafter indicated as $r_{on}$ and $r_{off}$ $\left(r_{on}<r_{off}\right)$ . Hence, don=R×(ron100) and doff=R×(roff100) . The FMFs and the values of their parameters are described in Table 1. These FMFs were extracted from the MATLAB fuzzy logic toolbox. Three traditional functions (denoted here as Shifting1, Shifting2 and Switching), which were taken from molecular dynamics studies (Loncharich and Brooks, 1989), were additionally used to compute membership degrees (see SI1-Section 1).

**TABLE 1:** Description, mathematical and parameters definitions of the fuzzy membership functions (FMFs) considered in this study.

Function	Definition	Parameters
S-shaped^s^	$\mu_{A}\left(x\right)=\{\begin{array}{cc} 0 & x\leq a\\ 2\times {\left(\frac{x-a}{b-a}\right)}^{2} & a\leq x\leq \frac{a+b}{2}\\ 1-2\times {\left(\frac{x-b}{b-a}\right)}^{2} & \frac{a+b}{2}\leq x\leq b\\ 0 & x\geq b \end{array}$	*x* = *d*_*io*_
• Parameter *a* defines the foot of the function, *b* defines its shoulder		*a* = *d*_*on*_
• It is related to the Z-shaped and PI-shaped FMFs		*b* = *d*_*off*_
Z-shaped^c^	$\mu_{A}\left(x\right)=\{\begin{array}{cc} 1 & x\leq a\\ 1-2\times {\left(\frac{x-a}{b-a}\right)}^{2} & a\leq x\leq \frac{a+b}{2}\\ 2\times {\left(\frac{x-b}{b-a}\right)}^{2} & \frac{a+b}{2}\leq x\leq b\\ 0 & x\geq b \end{array}$	*x* = *d*_*io*_
• Parameter *a* defines the shoulder of the function, *b* defines its foot		*a* = *d*_*on*_
• It is related to the S-shaped and PI-shaped FMFs		*b* = *d*_*off*_
PI-shaped^m^	$\mu_{A}\left(x\right)=\{\begin{array}{cc} 1 & x\leq a\\ 2\times {\left(\frac{x-a}{b-a}\right)}^{2} & a\leq x\leq \frac{a+b}{2}\\ 1-2\times {\left(\frac{x-b}{b-a}\right)}^{2} & \frac{a+b}{2}\leq x\leq b\\ 1 & b\geq x\leq c\\ 1-2\times {\left(\frac{x-c}{d-c}\right)}^{2} & c\leq x\leq \frac{c+d}{2}\\ 2\times {\left(\frac{x-d}{d-c}\right)}^{2} & \frac{c+d}{2}\leq x\leq d\\ 0 & x\geq d \end{array}$	*x* = *d*_*io*_
• Parameters *a* and *d* define the feet of the function, *b* and *c* define its shoulders		*a* = *d*_*on*_
• It is a product of a S-shaped function and a Z-shaped FMFs		*b*= (*d*_*on*_ *+ d*_*off*_)×0.45
		*c*= (*d*_*on*_ *+ d*_*off*_)×0.55
		*d* = *d*_*off*_
Triangular^m^	$\mu_{A}\left(x\right)=\{\begin{array}{cc} 0 & x\leq a\\ \frac{x-a}{b-a} & a\leq x\leq b\\ \frac{c-x}{c-b} & b\leq x\leq c\\ 0 & x\geq c \end{array}$	*x* = *d*_*io*_
• Parameters *a* and *c* define the feet of the function, *b* define its peak		*a* = *d*_*on*_
		*b*= (*d*_*on*_ *+ d*_*off*_) ×0.5
		*c* = *d*_*off*_
Trapezoidal^m^	$\mu_{A}\left(x\right)=\{\begin{array}{cc} 0 & x\leq a\\ \frac{x-a}{b-a} & a\leq x\leq b\\ 1 & b\leq x\leq c\\ \frac{d-x}{d-c} & c\leq x\leq d\\ 0 & x\geq d \end{array}$	*x* = *d*_*io*_
• Parameters *a* and *d* define the feet of the function, *b* and *c* define its shoulders		*a* = *d*_*on*_
		*b*= (*d*_*on*_ *+ d*_*off*_)×0.45
		*c*= (*d*_*on*_ *+ d*_*off*_)×0.55
		*d* = *d*_*off*_
Sigmoid-based	$\mu_{A}\left(x\right)=\frac{1}{1+e^{-a*\left(x-c\right)}}$	*x* = *d*_*io*_
• To open this FMF to the left or right, specify a negative or positive value for a, respectively		*c*= *(d*_*on*_ *+ d*_*off*_) ×0.5
• The magnitude of *a* defines the width of the transition area, and *c* defines the center of the transition area		Descending Sigmoid
• This function can be used in two modes: descending^c^ and ascending^s^		*a* = -1
		Ascending Sigmoid
		*a* = 1
Gaussian-based	$\mu_{A}\left(x\right)=e^{\frac{-{\left(x-c\right)}^{2}}{2\times a^{2}}}$	Descending Gaussian
• Parameter *a* is a measure of the width of the curve, and *c* defines the center of the curve		*a*=(*d*_*off*_ *-d*_*on*_) ×0.5
• This function can be employed in three ways: descending^c^, centralized^m^ and ascending^s^		*c* = *d*_*on*_
		Centralized Gaussian
		*a*=(*d*_*off*_ *-d*_*on*_) ×0.25
		*c*=(*d*_*on*_ *+ d*_*off*_) ×0.5
		Ascending Gaussian
		*a*=(*d*_*off*_ *-d*_*on*_) ×0.5
		*c* = *d*_*off*_
Generalized Bell-based	$\mu_{A}\left(x\right)=\frac{1}{1+{\left|\frac{x-c}{a}\right|}^{2b}}$	*b* = 2
• Parameter *a* defines the width of the curve, *b* controls the slope of the curve, and *c* is the center of the curve		Descending Bell
• This function can be employed in three modes: descending^c^, centralized^m^ and ascending^s^		*a*=(*d*_*off*_ *-d*_*on*_) ×0.5
		*c* = *d*_*on*_
		Centralized Bell
		*a*=(*d*_*off*_ *-d*_*on*_) ×0.25
		*c*=(*d*_*on*_ *+ d*_*off*_) ×0.5
		Ascending Bell
		*a*=(*d*_*off*_ *-d*_*on*_) ×0.5
		*c* = *d*_*off*_

Note: The superscripts *c*,*m* and *s* denote that the truncation function assigns more importance to amino acids close to the lower boundary (e.g., the center of the protein), middle region and upper boundary (e.g., surface of the protein) of the analyzed interval, respectively.

**SCHEME 1:**
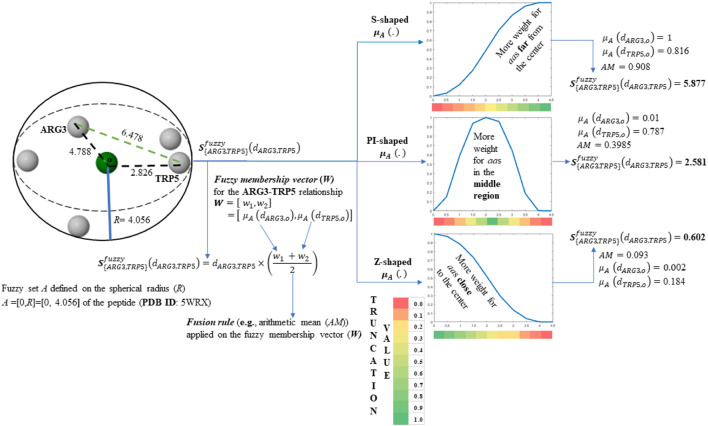
Tensor entry-level calculation corresponding to the ARG3-TRP5 interaction. The values of the obtained tensor entries (*z*_*35*_, *z*_*53*_) are in bold. The molecule employed consist of the first five-amino acid fragment of the peptide (PDB ID 5WRX) that is represented by its C_β_-atoms.

**FIGURE 1:**
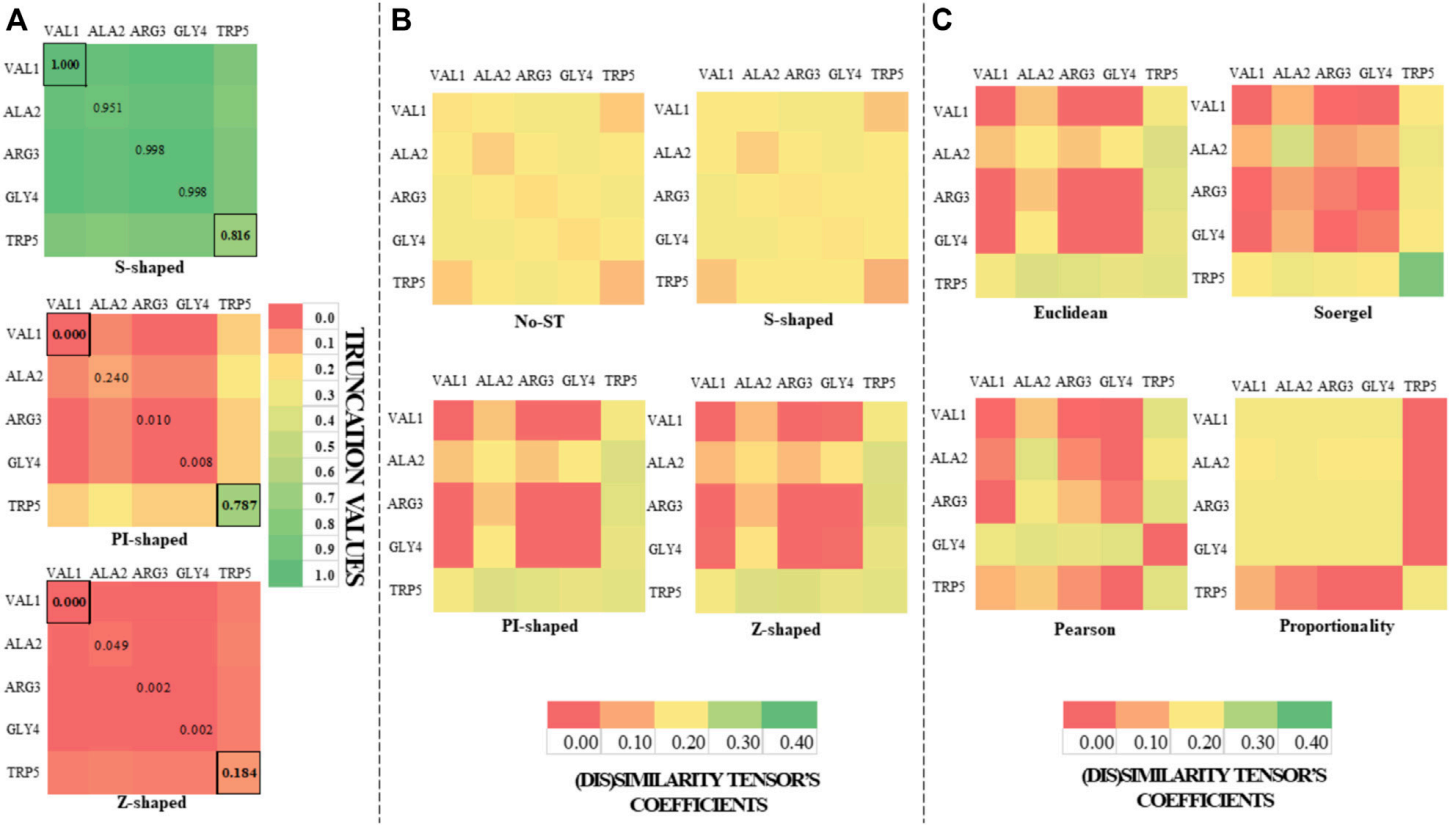
Color maps derived from the application of the S-shaped, PI-shaped and Z-shaped FMFs in the construction of two-tuple (5 × 5) spatial (dis)similarity tensors of order 1 to encode first five residues of the peptide (PDB ID 5WRX). The entries for the outermost (VAL1) and the innermost (TRP5) amino acids with respect to the geometrical center are bordered. All the FMFs consider an interval [0, *R*] = [0, 4.056], where *R* is the radius of the peptide. **(A)** Color maps of the truncation values derived from the application of the S-shaped, PI-shaped and Z-shaped FMFs in the construction of two-tuple spatial (dis)similarity tensors. **(B)** Color maps based on the coefficients of the mutual probability (MP) two-tuple non-truncated tensors (upper color maps), as well as MP two-tuple tensors truncated with different FMFs (the below ones). The tensors corresponding to Figures **(A,B)** were computed from the *Euclidean metric*; **(C)** Color maps based on the coefficients of the MP two-tuple tensors built from different (dis)similarity metrics and truncated with the PI-shaped FMF.

The FMFs listed on Table 1 allow the truncation of inter-amino acid interactions according to the proximity of *aas* with respect to a lower boundary $\left(d_{on}\right)$ , to an upper boundary $\left(d_{on}\right)$ , or to a middle region of the interval defined for the *A* fuzzy set. Thus, depending on the chosen FMF, the *aas* within a relationship will have different membership degrees in the truncation. Scheme 1 shows the workflow for the calculation of spherically truncated (dis)similarity matrices by using FMFs. In addition, Figure 1 shows three sets of color maps illustrating (Loncharich and Brooks, 1989): the biases of the S-shaped, PI-shaped and Z-shaped FMFs in the calculation of the membership degrees for the *aa*s belonging to the 5WRX (PDB ID) sub-peptide (first five residues) (see Figure 1A); (Mark and Nilsson, 2002) the effect of the three FMFs aforementioned in the truncation of a (dis)similarity two-way matrix, whose coefficients were calculated with the Euclidean metric (see Figure 1B); and (Todeschini et al., 2009) the effect of the method proposed in the truncation of different (dis)similarity two-tuple matrices by considering a same FMF (see Figure 1C). The *A* fuzzy set used both in Figure 1 and Scheme 1 is defined on the spherical radius of the 5WRX peptide: *A* = [0, 4.056]. The *o* geometric center of the peptide (*x*_o_, *y*_o_, *z*_o_) is equal to (3.139, −0.960, 2.148).

On the one hand, it can be noted from Figure 1A and Scheme 1 that the S-shaped FMF assigns more importance to *aas* far from the geometrical center of the 5WRX peptide [e.g., $\mu_{A}\left(d_{VAL1,O}\right)=\mu_{A}\left(4.06\right)=1$ ; $\mu_{A}\left(d_{TRP5,O}\right)=\mu_{A}\left(2.83\right)=0.816$ ]. Consequently, it can be noted from Figure 1B that the color map of the non-truncated matrix (No-ST) is quite like the one obtained from the S-shaped FMF because, at least for the interval and peptide considered, the S-shaped FMF generates membership values with tendency to 1. Thus, this FMF does not almost have effect in the truncation of the original matrix (No-ST). On the other hand, it can also be observed in Figure 1A and Scheme 1 that the Z-shaped FMF gives more importance to *aa*s near to the center [*e.g.,* $\mu_{A}\left(d_{VAL1,O}\right)=\mu_{A}\left(4.06\right)=0$ ; $\mu_{A}\left(d_{TRP5,O}\right)=\mu_{A}\left(2.83\right)=0.184$ ], whereas the PI-shaped FMF assigns the highest membership values to *aa*s within the middle region of the interval analyzed [*e.g.,* $\mu_{A}\left(d_{VAL1,O}\right)=\mu_{A}\left(4.06\right)=0$ ; $\mu_{A}\left(d_{TRP5,O}\right)=\mu_{A}\left(2.83\right)=0.787$ ]. Thus, the color maps of the matrices truncated with the PI-shaped and Z-shaped FMFs (see Figure 1B) are rather different from the color map of the non-truncated matrix. It can also be noted in Figure 1B, the similarity between the color maps corresponding to the PI-shaped and Z-shaped FMFs, which confirms that the *aa*s in the peptide considered are distant from its geometrical center. Lastly, it can be observed from Figure 1C the color maps obtained when several metrics are used to compute the (dis)similarity matrices.

**SCHEME 2:**
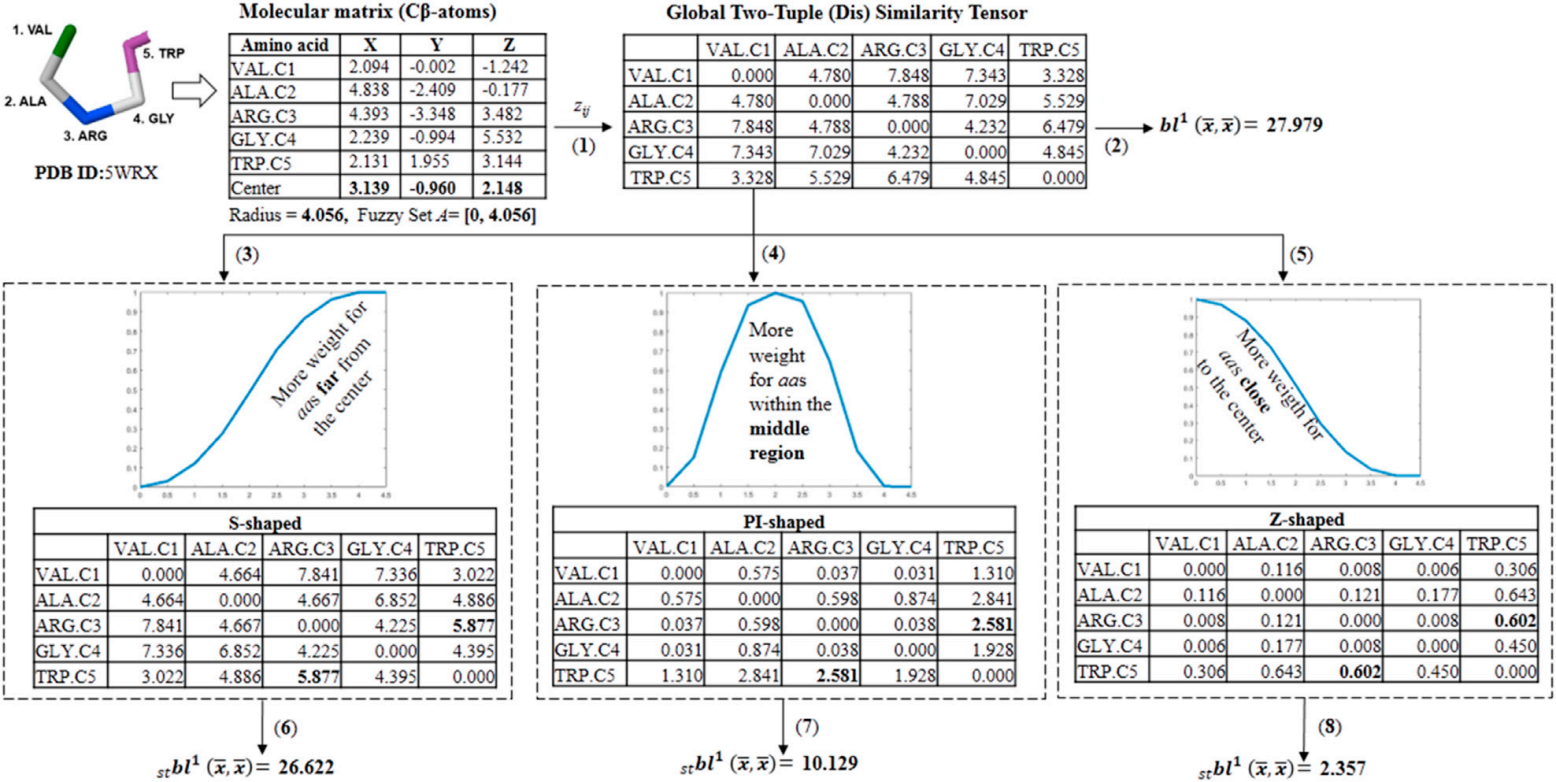
Workflow followed in the calculation of different fuzzy spherically truncated two-tuple non-stochastic tensors and their corresponding two-linear (quadratic) PDs by using the for the five-amino acid fragment of the peptide **5WRX**. (1) The global two-tuple (dis) similarity tensor (^D^Z) is computed from the Euclidean metric. (2) The classical quadratic PD is computed from ^D^Z by using the electronic charge (ECI) as a weighting scheme. (3,4 and 5) Fuzzy spherically truncated two-tuple tensors (ST-^D^Z) obtained from ^D^Z by using the S-shaped, PI-shaped and Z-shaped FMFs, respectively. (6,7 and 8) The fuzzy spherically truncated quadratic PDs are calculated from ST-^D^Z.

**SCHEME 3:**
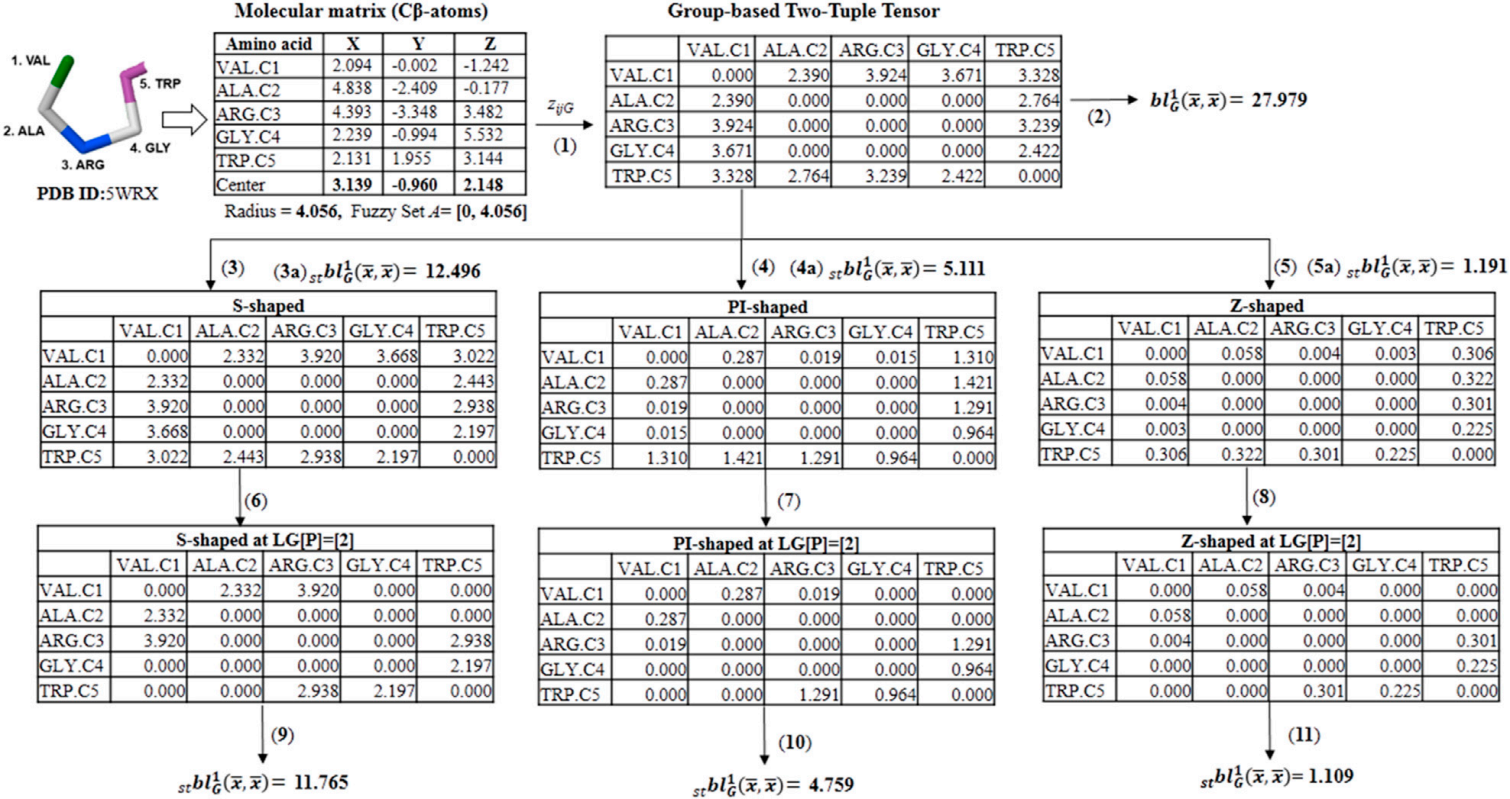
Illustration calculation of different group-based fuzzy spherically truncated two-tuple non-stochastic tensors and their corresponding descriptors by using the for the five-amino acid fragment of the peptide **5WRX**. (1) The group-based (dis)similarity two-tuple tensor (^D^Z_G_) is computed with the Euclidean distance and the beta-sheet favoring (FBS) group. (2) The classical group-based two-linear (quadratic) PDs is determined from ^D^Z_G_ and the property electronic charge (ECI). (3, 4 and 5) Spherically truncated group-based two-tuple tensors (ST- ^D^Z_G_) obtained from Z_G_ by applying the S-Shaped, PI-Shaped and Z-Shaped FMFs, respectively. (3a, 4a and 5a) Spherically truncated quadratic PDs obtained from ST- ^D^Z_G_. (6, 7 and 8) Group-based spherically-truncated at topological *lag* [p] = [2] (LG[p]-ST-Z_G_) two-order tensors computed from the ST-^D^Z_G_ tensors. (9, 10 and 11) Spherically-truncated quadratic PDs at topological *lag* [p] = [2] obtained from LG[p]-ST- ^D^Z_G_.

The truncation method explained above is only applied on the two-tuple (ℤ(G)1D) and *t*hree-tuple (ℤ(G)1T) spatial-(dis) similarity matrices of order 1, in order to obtain the fuzzy spherically truncated two-tuple (ST-ℤ(G)1D) and three-tuple (ST-ℤ(G)1T) spatial-(dis) similarity tensors of order 1. Then, on the ST-ℤ(G)1D and ST-ℤ(G)1T matrices, all the theoretical generalizations defined for the original spatial-(dis) similarity tensors (Terán et al., 2019; Marrero-Ponce et al., 2020) can also be applied to compute the *k*th (k≥1) fuzzy spherically truncated two-tuple (ST-ℤ(G)kD) and three-tuple (ST-ℤ(G)kT) spatial-(dis) similarity matrices (see Schemes 2, 3). In this way, the *k*th fuzzy spherically truncated two-tuple (ST-ℤ(G)aa,kD) and three-tuple (ST-ℤ(G)aa,kT) spatial-(dis) similarity aa-level matrices can be derived for each *aa* into a protein, with the purpose of calculating the *k*th fuzzy *s*pherically truncated two-linear (ST-L(G)aa,kD) and three-linear (ST-L(G)aa,kT) *aa-level* MuLiMs-MCoMPAs PDs in a similar way to Eqs 1, 2. Each *aa-level* MuLiMs-MCoMPAs 3D-PD is an entry in the corresponding LAI vector, on which one or several aggregation operators can be applied to compute the *k*th global fuzzy spherically truncated two-linear (or three-linear) MuLiMs-MCoMPAs 3D-PDs. These fuzzy spherically truncated 3D-PDs for proteins will encode biochemical information corresponding to geometrical relationships among *aa*s in a protein, by considering the closeness of *aas* either to the geometrical center, or to a middle region, or to the surface of a protein.

## 4 Exploratory analysis

### 4.1 Spherical radius-based amino acid distribution analysis

This section analyzes the *aas’* distribution considering their distances respect to the protein’s geometrical center. The evaluated set considered 152 heterogeneous and representative proteins, which contain between 50 and 753 *aa*s (Fleming and Richards, 2000). These proteins were represented by using the four spatial protein representation available in the MuLiMs-MCoMPAs software: alpha (CA), beta (CB), amide-bond (AB) carbon atoms, and average of the spatial coordinates of all the heavy atoms (AVG). For each studied protein, 10 intervals (bins) were defined, where each interval represents a 10% of the length of the spherical radius of the proteins considered, being 0 the geometric center. The intervals were defined as follows [0,10), [10,20), [20,30), [30,40), [40,50), [50,60), [60,70), [70,80), [80,90) and [90,100].

**FIGURE 2:**
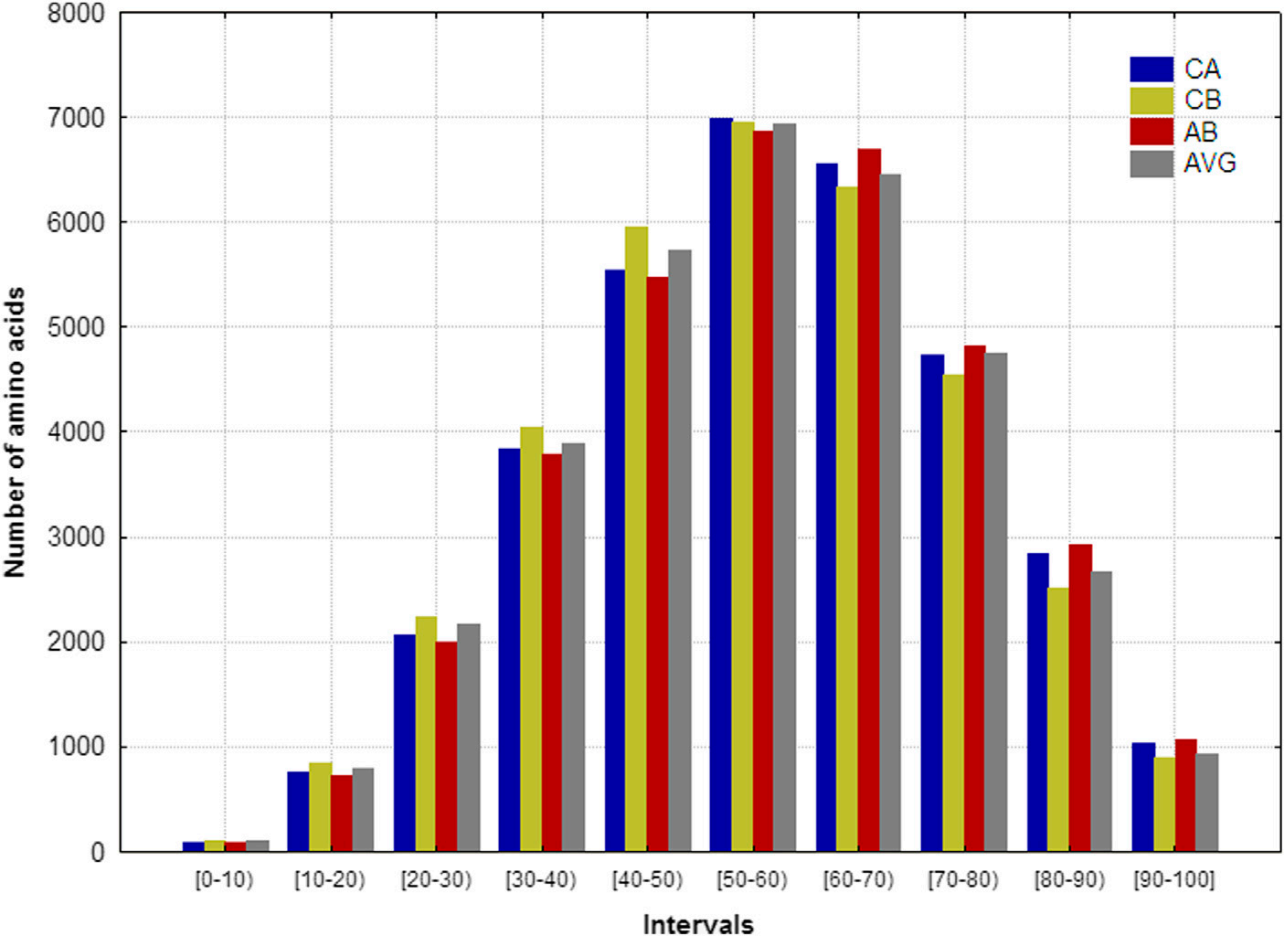
Frequency distributions of the number of amino acids that fall into the 10 intervals defined on the spherical radius for the 152 heterogeneous proteins. The proteins were represented according to the coordinates of: alfa (CA), beta (CB), amide-bond (AB) carbon atoms, as well as the average of the spatial coordinates all heavy atoms (AVG).

Figure 2 shows the *aa*s’ frequency distributions that fall into the 10 intervals analyzed for each representation. Notably, the four spatial representations’ frequency distributions are very similar, suggesting that these frequency distribution remains invariant regardless of the representation employed. The maximum frequencies are placed between 30% and 80% of the *R* values, pointing out that the majority of *aa*s belongs to the middle region of the “artificial” sphere where each protein is embedded. The second largest amount of *aa*s was located between 80% and 100% of the *R* values (surface region of the sphere), whereas the remaining *aa*s are near to the geometric center. These results are in correspondence with the ones obtained elsewhere (García-Jacas et al., 2019).

Since every *aa* was assigned a “membership degree” according to the proximity to a protein’s “spherical region” of interest, we can justify the use of FMFs to truncate inter amino acid relationships as a tool to extract information from the protein’s spatial arrangement.

### 4.2 Exploratory analysis of the truncation functions based on descriptive statistics and cluster analysis

An exploratory analysis of the traditional functions and FMFs chosen in this study is performed using the previous dataset (152 proteins) (Fleming and Richards, 2000). The proteins were represented only using the CB representation. For every *aa* that belonged to each of the 152 proteins, 16 “membership degrees” (one per truncation function) were computed using the interval [0, *R*] as the fuzzy set *A*. The computed membership values were arranged into a matrix of 34,584 rows (total number of *aa*s analyzed) and 16 columns (fuzzy and traditional functions). For the membership values obtained from each truncation function, the minimum (Min), maximum (Max), arithmetic mean (Ave), and standard deviation (Std) statistical parameters were calculated. These statistics are listed in Table 2.

**TABLE 2:** Descriptive statistics for the “membership values” derived from the 16 truncation functions for the dataset of 152 non-homologous proteins.

Truncation function	Min	Max	Am	Std
Shifting1	0	0.999	0.473	0.251
Shifting2	0	0.954	0.231	0.172
Switching	0	0.007	0.001	0.0007
S-shaped	0.001	1	0.581	0.267
Z-shaped	0	0.999	0.419	0.267
PI-shaped	0	1	0.788	0.275
Triangular	0	1	0.682	0.226
Trapezoidal	0	1	0.746	0.239
D-Sigmoid	0	1	0.388	0.423
A-Sigmoid	0	1	0.612	0.423
D-Gaussian	0.135	0.999	0.543	0.202
C-Gaussian	0.135	1	0.781	0.219
A-Gaussian	0.148	1	0.665	0.198
D-Bell	0.059	1	0.447	0.269
C-Bell	0.059	1	0.763	0.287
A-Bell	0.064	1	0.609	0.278

The first observation relates to the Switching traditional function, which is a notable outlier, since it yields low values compared to the other functions (Max = 0.007). Another outcome shown in Table 2 suggests that the PI-shaped, C-Gaussian, C-Bell, and Trapezoidal FMFs yield high membership values since they present arithmetic mean values greater than 0.7. This behavior relates to the type of FMFs associated to these functions. These FMFs tend to assign the highest membership degrees to *aa*s in the middle region, which mostly approximates to a spherical region. The D-Sigmoid and A-Sigmoid FMFs present the most scattered membership values (both with standard deviation greater than 0.40), whereas the A-Gaussian, Shifting2, and Switching functions have the least scattered membership values (standard deviation less than 0.2).

**FIGURE 3:**
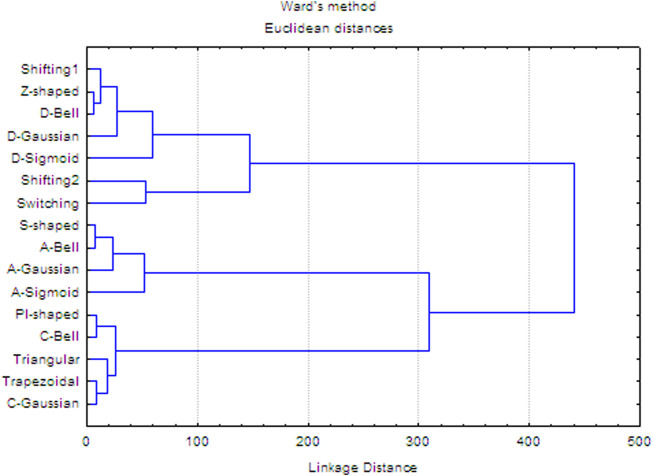
Hierarchical cluster analysis of the 16 truncation functions using Ward’s method.

Moreover, diversity relationships between the fuzzy and traditional functions were explored using hierarchical clustering with the Ward agglomeration method (Jain and Dubes, 1988; Rivera-Borroto et al., 2011). To perform this analysis, a 16 × 16 matrix was built, where each matrix-entry is the Euclidean distance between the membership degrees corresponding to each pair of truncation functions (see SI1-Table 1). The dendrogram obtained is shown in Figure 3, where at a cutoff distance equal to 100 (20% of the linkage distance), four clusters can be identified. These clusters explain the grouping of the 16 truncation functions according to their biases in the calculation of membership degrees. Considering the first two clusters (from top-to-down), they represent functions with a tendency to assign the largest membership degrees to *aa*s close to the geometrical center (e.g., Z-shaped, Shifting2). The third cluster represents functions with bias toward the amino acids placed far from the geometrical center (e.g., S-shaped, A-Gaussian, A-Sigmoid, and so on), while the fourth cluster represents functions with the propensity to give more importance to *aa*s in the middle region (e.g., PI-shaped, Triangular, C-Gaussian and so on). These results evidence that the studied traditional and fuzzy functions are diverse, and therefore they should provide orthogonal chemical information regarding the one codified by other reported 3D-PDs.

## 5 Variability and linear independence analyses of the fuzzy spherically truncated mulims-mcompas descriptors

The sensitivity of an PD to structural or conformational changes, also known as variability, is one of their desirable features when calculating them (Randić, 1995). This variability has been evaluated through the Shannon’s entropy (SE) calculation. According to this theory, a high-entropy PDs provide higher variability and therefore these PDs are relevant for modeling studies.

Another desirable feature for a novel PDs refers to orthogonality with respect to other PDs (Randić, 1995). A mean to measure this relies on the Principal Components Analysis (PCA) technique (Bro and Smilde, 2014; Lever et al., 2017). PCA is probably the most popular multivariate statistical technique, and it is used by almost all scientific disciplines. PCA is an unsupervised learning approach and is like clustering (it finds patterns). This method analyzes a data table representing observations described by several dependent variables (here PDs), which are, in general, inter-correlated. The PCA goal is to extract the important information from the data and to express this information as a set of summary indices called principal components. That is, this analysis transforms the original variables in orthogonal variables (principal components) from each other (Somorjai and John, 2010). The main applications of factor analysis techniques are: 1) to reduce the number of variables, and 2) to detect structure in the relationships between variables, that is to classify variables. Thus, these factors capture most of the “essence” of these PDs because they are a linear combination of the original items. Because each consecutive factor is defined to maximize the variability that is not captured by the preceding factor, consecutive factors are independent of each other. Put another way, consecutive factors are uncorrelated or orthogonal to each other. The first factor obtained is generally more highly correlated with the variables than the other factors. This is to be expected because these factors are extracted successively and will account for less and less variance overall. Finally, some of the most important conclusions that can be drawn from a factor analysis that will be of great usefulness in the present report are the following: 1) variables with a high loading in the same factor are interrelated and will be the more so the higher the loadings, and 2) no correlation exists between variables having nonzero loadings only in different factors (Bro and Smilde, 2014; Lever et al., 2017). These are the principal ideas that permit interpreting the factor structure obtained using the factor analysis as a classification method. Specifically, after mean-centering and scaling to unit variance, the data set is ready for computation of the first principal component (PC1). The correlation between a component and a variable (here PDs) estimates the information they share. In the PCA framework, this correlation is called a loading. The variables (PDs) can be plotted as points in the component space using their loadings as coordinates. This representation differs from the plot of the observations: The observations are represented by their projections, but the variables are represented by their correlations. Variables with high loading values for a principal component means a high contribution to this component and vice versa, so, variables with low PC loadings are less important for this component. Some variables have a positive sign (a positive important contributions to this component), and other depict negative loading, show a negative important contribution to the same component. The sign only indicates that variable has a negative correlation to that component. Really, what matters is the magnitude of the value, and the larger it is, the greater the contribution to that component. Variables contributing similar information are grouped together, for instance, with high loading (high correlation) to same component. When they are positively correlated with principal component (when the numerical value of one variable increase or decreases, the numerical value of the other variable tends to change in the same way). When variables are negatively (“inversely”) correlated with same principal factor, meaning that when these variable increases, the other with positive loading decreases, and vice versa. In conclusion, the modulus loading value of each variable it is the rally important because it indicates the strength of relation of variable with component (principal component loadings is the correlation of a component and a variable, and geometrically express the orientation of the model plane in the K-dimensional variable space (Bro and Smilde, 2014; Lever et al., 2017).

The factor analysis was performed with the STATISTICA software, and “varimax normalized” was used as a rotational strategy to obtain the factor loadings from the PCA. The goal of this rotational procedure is to obtain a clear pattern of loadings, that is, factors that are somehow clearly marked by high loadings for some variables and low loadings for others. The “varimax normalized” is the method that is most commonly used as “varimax” rotation. This rotation strategy is aimed at maximizing the variances of the squared normalized factor loadings (row factor loadings divided by squared roots of the respective communalities) across variable for each factor. This strategy makes the structure of factors pattern as simple as possible, permitting a clearer interpretation of the factors without loss of orthogonality between them.

Consequently, this section aims to evaluate the variability and orthogonality of the fuzzy spherically truncated MuLiMs-MCoMPAs PDs using the previously presented set of 152 proteins (Fleming and Richards, 2000). These evaluations were performed using the IMMAN (Urias et al., 2015) and STATISTICA software (v8.0), respectively.

### 5.1 Shannon Entropy-based variability of the two-linear MuLiMs-MCoMPAs 3D-MDs based on fuzzy membership functions

The SE-based variability analysis was performed by creating a project comprised of 64 traditional two-linear MuLiMs-MCoMPAs 3D-PDs (See Reference 20 for more details about projects). From this starting project, 16 additional projects (one per truncation function) were created to compute the corresponding truncated MuLiMs-MCoMPAs PDs. Each out of 16 projects corresponds to the use of a single traditional function (TF) or a FMF. For each traditional function or FMF, various intervals (*r*_on_-*r*_off_) were examined. For the Shifting1 and Shifting2 functions 10 intervals were analyzed (see SI2_A), whereas for the Switching function and FMFs 15 intervals were evaluated. The 3D-PDs defined in these projects were computed using the MuLiMs-MCoMPAs software (Contreras-Torres et al., 2019). The truncated 3D-PDs calculated with the same function (different intervals) were merged into a single dataset. Then, the merged 16 datasets were analyzed performing a SE-based variability study through the IMMAN software (see SI2_B) (Urias et al., 2015). The SE values computation considered that the number of proteins in the analyzed dataset (152) was used as binning scheme. So, the maximum SE for each PD is equal to log_2_(152) = 7.24 bits.

**FIGURE 4:**
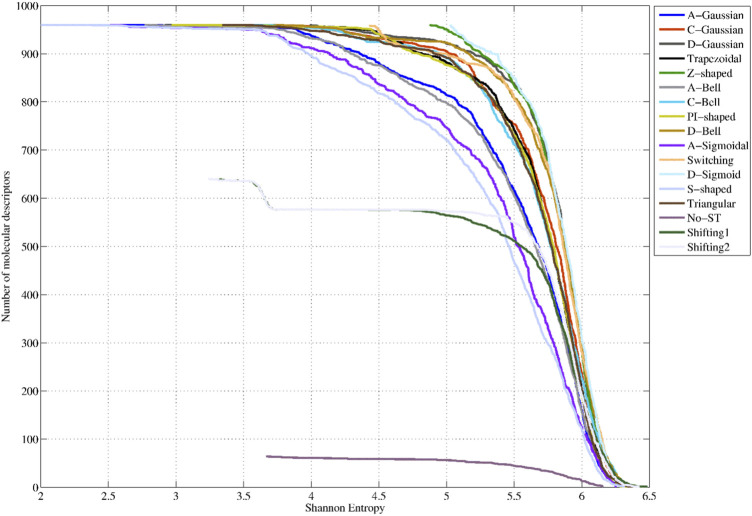
Shannon Entropy distributions corresponding to the 64 classical 2-linear 3D-PDs (denoted as No-ST), the 640 2-linear 3D-MDs truncated Shifiting1 and Shifting2 (traditional functions, TFs), the 960 3D-MDs truncated with the Switching (traditional function, TFs) and the 960 3D-PDs based on each fuzzy membership functions (FMFs).

Figure 4 shows the SE distributions corresponding to the 64 non-truncated 3D-PDs, the 640 3D-PDs truncated with the Shifting1 and Shifting2 TFs, the 960 3D-PDs truncated with the Switching TF, and the 960 3D-PDs computed with each FMF (see SI2_B). It can be observed that the best SE distributions correspond to the PDs truncated with the D-Sigmoid, Z-Shaped, Switching, D-Gaussian and D-Bell functions. These obtained PDs yielded SE values greater than 79% of the maximum SE. Note that the truncation functions mentioned above tend to compute the highest membership values for the *aa*s near to the geometrical center of the protein.

Intermediate SE distributions are obtained by the 3D-PDs based on the C-Gaussian, Trapezoidal, C-Bell, PI-shaped, Triangular, Shifting2 and Shifting1 functions, which yielded average SE values between 77% and 79% of the maximum SE. This set of functions, excepting for the Shifting2 and Shifting1 functions, tend to assign the highest weights to *aa*s located in middle regions. The lowest performances are attained by the 3D-MDs based on the A-Gaussian, A-Bell, A-Sigmoidal and S-shaped FMFs, considering their average SE values lower than 77% of the maximum SE. It is remarkedly that these truncation functions tend to assign more importance to distanced *aa*s from the geometrical center. These outcomes confirm that the application of FMFs to truncate inter-amino acid relationships while encoding 3D-PDs can enrich their information content, evidencing the feasibility of the truncation approach.

**FIGURE 5:**
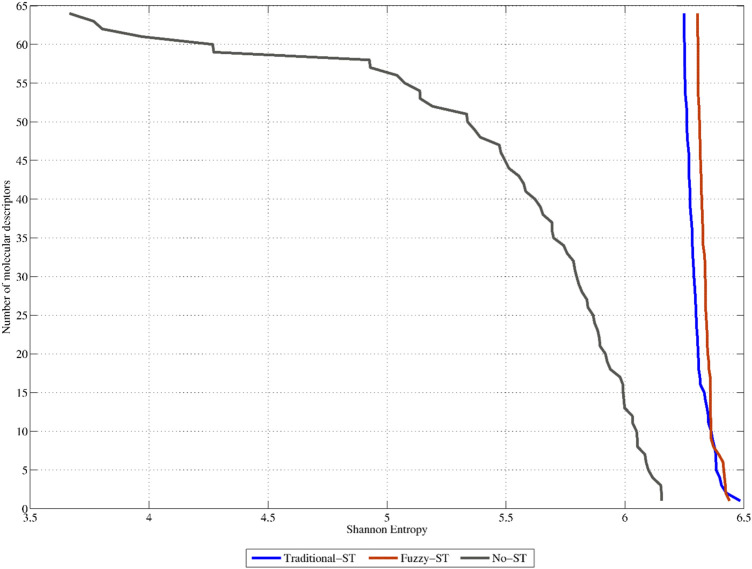
Shannon Entropy distributions corresponding to the 64 top-ranked classical 2-linear 3D-PDs (denoted as No-ST), the 64 top-ranked 2-linear 3D-MDs truncated with traditional functions (Traditional-ST), and the 64 top-ranked 2-linear 3D-MDs truncated with fuzzy membership functions (Fuzzy-ST), respectively.

The previous assertion is also demonstrated in Figure 5. This figure shows the SE distribution of the 64 non-truncated 3D-PDs (termed No-ST), and of their truncated counterparts (64 best-ranked PDs truncated with the TFs (termed Traditional-ST)) and FMFs (termed Fuzzy-ST), respectively (see also SI2_B). It can be observed that the 3D-PDs truncated with FMFs display the best SE distribution since all their respective values are beyond 6.17 bits, 85.22% of the maximum SE. These SE values are higher than the ones obtained by the non-truncated 3D-PDs and 3D-PDs truncated with the TFs. In summary, it can be stated that all the sets of truncated 3D-MDs contain high variability, suggesting that the truncation-based calculations encode relevant information, mainly by using FMFs.

### 5.2 Linear independence of the two-linear MuLiMs-MCoMPAs 3D-MDs based on fuzzy membership functions

This section assesses linear independence between the non-truncated and truncated 3D-PDs. To this end, the 20 best-ranked non-truncated and truncated PDs, according to their SE values, were extracted from each TF and FMF files; and then they were merged into a single dataset. The resulting dataset contains 340 PDs, which were used as input for the PCA method (see SI-3.1). By applying the PCA method, 15 principal components that collectively explain 87.90% of the cumulative variance were obtained (see SI-3.2). Those PDs strongly loaded (Loading value≥0.7) in a same component are linearly dependent, whereas PDs strongly loaded in different components are linearly independent. The inspection of these components reveals that the truncated 3D-PDs are exclusively loaded in 11 principal components (i.e., PC1, PC2, PC4-PC8, PC10-PC13; 74.14% of the cumulative variance), indicating that the new truncation method contributes to codifying linearly independent information with respect to non-truncated 3D-PDs.

Out of 11 PCs corresponding to the truncated 3D-PDs, three are exclusively populated of the 3D-PDs truncated with FMFs, which support the novelty of the proposed truncation approach presented in this study. In PC5, the 3D-PDs truncated with the C-Bell, C-Gaussian, PI-shaped, Trapezoidal and Triangular FMFs are loaded while PC12 contains 3D-PDs truncated with the A-Bell, A-Gaussian, C-Bell, C-Gaussian, D-Bell, D-Gaussian, D-Sigmoid, PI-shaped, Trapezoidal and Triangular FMFs; and PC13 presents loadings for the 3D-PDs truncated with the A-Gaussian, A-Sigmoid and S-shaped FMFs. Focusing the attention on PC5, the truncated 3D-PDs strongly loaded on it, are those giving more importance to *aa*s belonging to the middle region (e.g., PI-shaped), whereas the PDs truncated with FMFs assigning high weights to aas far from the geometric center (e.g., S-shaped) are loaded in PC13. These findings evidence that the FMFs are suitable smoothing functions to truncate inter-amino acid relationships, at allowing to codify orthogonal structural information regarding the one encoded by the TFs (see SI-3.2).

The PCA method also revealed redundancy in the information encoded by some of 3D-PDs truncated with FMFs and TFs. This redundancy can be because some FMFs give more importance to *aa*s closer to the geometric center of the protein (e.g., Z-shaped) in a similar way to the TFs. For instance, PC2 presents 3D-PDs truncated with the D-Bell, D-Gaussian, D-Sigmoid, Z-shaped, Shifting1 and Shifting2 functions.

This suggests that any of the previous functions can be applied without distinction. Moreover, PC10 is exclusive for 3D-PDs based on the Switching TF, whereas that PC3 and PC9 hold loadings of non-truncated and truncated 3D-MDs; the last ones mainly based on functions that assign more weight to *aa*s far from the geometrical center (e.g., S-shaped). From this analysis, models with better predictive ability can be expected from 3D-PDs truncated with TFs and/or FMFs biased to the center (and middle region) of the protein.

## 6 Application of the spherically truncated MuLiMs-MCoMPAs descriptors to the prediction of protein folding rate and SCOP structural classes

### 6.1 Biochemical datasets

Protein folding is the one of most relevant problems in molecular biology and modern biophysics. It emerged more than 5 decades ago when Anfinsen showed that proteins spontaneously reach their native structures *in vitro* (Anfinsen, 1973). With the advent of computers, the prediction of the proteins’ folding rate became something attainable (Plaxco et al., 1998; Makarov et al., 2002; Gromiha, 2003; Ruiz-Blanco et al., 2015). There are several proteins sets available for testing the predicting ability of new descriptors (Ouyang and Liang, 2008; Ruiz-Blanco et al., 2015). In this study for the regression section, we chose a training set comprised of 80 proteins (PDB ID 2BLM was omitted due to low resolution, only contains the trace of alpha carbon atoms), while the test set is comprised of 16 proteins.

When considering protein related classification problems, the knowledge of protein structural classes plays a significant role in understanding protein folding process and function (Chou, 2005). In this study, we chose a well know protein structural discrimination set which includes 204 cases that are grouped according to the major SCOP classes (52 all-α, 61 all-β, 45 α/β and 46 α+β). This set was divided into training and test sets, (see Section 3.1 in Marrero-Ponce et al. (2020)). The training set contains 149 proteins (38 all-α, 48 all-β, 29 α/β and 34 α+β), whereas the test set collects 55 proteins (14 all-α, 13 all-β, 16 α/β and 12 α+β). It is important to indicate that these datasets (both folding rate and SCOP structural) were previously used to assess the No-ST (traditional) MuLiMs-MCoMPAs PDs (Terán et al., 2019; Marrero-Ponce et al., 2020), so comparability of results is guaranteed.

### 6.2 Truncation functions to enhance the predictive ability of traditional 3D-MDs in the protein folding rate modeling

The truncation method proposed was applied on the two-linear traditional 3D-PDs included in a previously developed model to predict protein folding rate (see Eq. 13 and Table 3 in Marrero-Ponce et al. (2020)). This model, hereafter reference model, was built with four two-linear traditional 3D-PDs by using the Multiple Linear Regression (MLR) method. From these four traditional 3D-PDs, their corresponding truncated versions based on FMFs and TFs were computed, to build the predictive models The 3D-PDs truncated with each smoothing function were merged with the non-truncated 3D-PDs into a single dataset. So, 16 datasets of 3D-PDs (one per smoothing function) were obtained, containing a total of 44 types of 3D-PDs (4 traditional 3D-PDs and 40 truncated PDs). These descriptor datasets were employed as input to develop the MLR models to predict folding rate. MLR models based only on truncated 3D-PDs were also built. The MLR models were developed using the Genetic Algorithm (GA) – MLR wrapper through the MobyDigs software (Todeschini et al., 2003).

The GA was set up with the default parameters. The allowed variables were limited up to four to ensure comparability with the reference model. The built model yielded external correlation coefficients Q2ext = 72.77% and Q2extw/o = 87.5% at including and removing outliers, respectively. This comparison took as reference the correlation value attained by the previously reported model (No-ST) without omitting outliers (Q2ext = 72.77%), otherwise it will be explicitly indicated.

**FIGURE 6:**
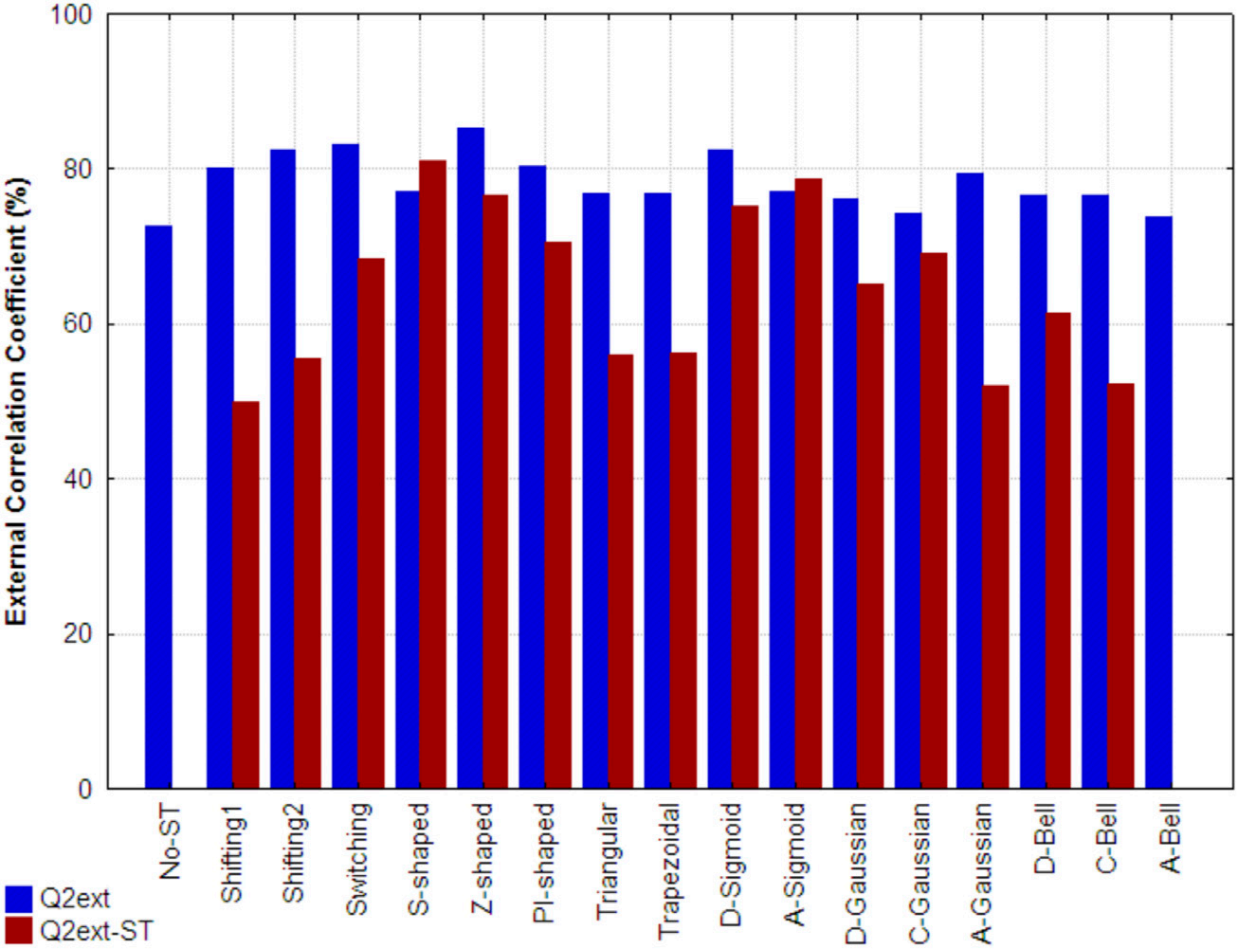
External correlation coefficient attained, on the folding rate set, by the reference model developed with classical 3D-PDs (indicated as No-ST), by the best model developed from each set of truncated 3D-PDs combined with the classical 3D-PDs (Q^2^_ext_) and by the best model based only on truncated 3D-MDs (Q^2^_ext_-ST). Each set of truncated 3D-PDs employs a traditional function or a fuzzy membership function. All the models were built using the MLR technique.

Figure 6 shows the external correlation coefficients of the best models developed using the non-truncated (traditional) and truncated 3D-PDs (Q2ext) for each respective function, as well as the ones obtained by the best models built up only from truncated 3D-PDs (Q2ext-ST). The models that include mixtures of non-truncated and truncated 3D-MDs yielded Q2ext values between 73% and 86%. All these values are higher than the one obtained by the reference model (indicated as No-ST). In particular, the best results correspond to the Z-shaped (Q2ext = 85.27%), Switching (Q2ext = 83.07%), D-Sigmoid (Q2ext = 82.5%), Shifting2 (Q2ext = 82.37), PI-shaped (Q2ext = 80.4%), Shifting1 (Q2ext = 80.04%), A-Gaussian (Q2ext = 79.46), A-Sigmoid (Q2ext = 77.16) and S-shaped (Q2ext = 77.01%) functions, yielding improvements of 12.5%, 10.3%, 9.7%, 9.6%, 7.6%, 7.27%, 6.69%, 4.39% and 4.24% with regard to the performance obtained by the reference model, respectively. Among the functions mentioned above, there are FMFs biased to the center (e.g., Z-shaped), middle region (e.g., PI-shaped) and surface (e.g.*,* A-Gaussian) of the protein, supporting its application to the truncation of inter-amino acid interactions.

Regarding the models uniquely built with the truncated 3D-PDs, the best results correspond to the S-shaped (Q2ext-ST = 80.94%), A-Sigmoidal (Q2ext-ST = 78.68%), Z-shaped (Q2ext-ST = 76.68%) and D-Sigmoid (Q2ext-ST = 75.13%) FMFs. These models outperform the reference model. It should also be pointed out that the result attained by the model based on the S-shaped FMF is even better than its analog built by mixing the non-truncated and truncated 3D-PDs. The models created with the remaining FMFs showed poor performance.

In general, the best models including 3D-PDs truncated with FMFs except the A-Gaussian type, provided acceptable predictions when non-truncated 3D-PDs were excluded. By contrast, the models based on the 3D-PDs truncated with TFs (i.e., Shifting1, Shifting2 and Switching) were highly sensitive when the non-truncated 3D-MDs were omitted, yielding poor correlations (30.18%, 26.89% and 14.61%).

So far, it has been demonstrated that the use of truncated 3D-PDs increases the predictive power of the reference model for folding rate prediction. It was demonstrated that the combination of non-truncated 3D-PDs and their corresponding truncated versions with either one of the FMF or TF classes yield better correlations than the exclusive use of non-truncated 3D-PDs. However, further improvements in protein rate folding modeling could be achieved by combining 3D-PDs truncated from different FMFs or TFs? In this sense, both non-truncated 3D-PDs and truncated 3D-PDs corresponding to the best smoothing functions (i.e., Shifting1, Shifting2, Switching, Z-shaped, D-Sigmoid, A-Sigmoid, PI-shaped and A-Gaussian) were merged into a single set. Then, MLR “hybrid models” were built by employing the same GA configuration. The statistical parameters of the best “hybrid models” and their corresponding MLR equations are listed in Table 3 (ID: M2, M3 and M4) and SI-4, respectively. These models displayed external correlations beyond 83%, surpassing the one obtained by the reference model. Notably, the highest external correlation attained (Q2ext = 86.45%, see Table 3, ID: M2), not only overcome the one attained by the models including a single truncation function class, but also it is comparable to the one attained by the reference model after outlier exclusion (Q2extw/o = 87.5%). This outcome indicates that higher robustness and predictive abilities can be achieved when PDs truncated with diverse smoothing functions are included in the model. In detail, the smoothing function-pairs used in the “hybrid models” are Switching/D-Sigmoid, Switching/Z-shaped and Shifting2/PI-shaped, being the Switching/Z-shaped the one that allowed the best performance. These findings suggest that the combinations of different 3D-PD classes (non-truncated and truncated by different functions) can enhance 3D protein structure characterization and consequently structure/function prediction models.

**TABLE 3:** Statistical parameters of the best models built in this study for the prediction of protein folding rates from truncated and classical MuLiMs-MCoMPAs 2-linear PDs.

Model ID	Number of MDs (based on FMFs-TFs)	R^2^	Q^2^_loo_	Q^2^_boot_	SDEP	Q^2^_ext_	a(Q^2^)	SDEP_ext_	Q^2^_extw/o_	SDEP_ext w/o_
M1	4(1–0)	77.94	74.78	74.25	2.16	85.27	−0.126	1.147	—	—
M2	4(1–1)	79.02	76.02	75.52	2.11	83.99	−0.128	1.195	—	—
M3	4(1–1)	78.04	74.89	74.07	2.16	86.45	−0.145	1.1	—	—
M4	4(1–1)	77.26	74.57	73.78	2.17	83.80	−0.131	1.2	—	—
M5	4(3–1)	80.21	77.28	76.59	2.06	75.05	−0.119	1.49	95.82	0.61

### 6.3 Truncation method to enhance the accuracy of structural class prediction

This section analyzes the effect of the smoothing functions on the predictive ability of the truncated two-linear MuLiMs-MCoMPAs 3D-PDs in the modeling of SCOP structural classes. To this purpose, QSAR models including 3D-PDs truncated with FMFs and TFs were developed. In detail, the effect of the truncated 3D-PDs on the prediction ability of a reported model for this application (see Table 6 in Marrero-Ponce et al. (2020)) was evaluated. Likewise, to the previous section, the non-truncated 3D-PDs belonging to the previously reported model, hereafter reference model, were used as starting point to compute the corresponding non-truncated versions. In particular, the reference model for the SCOP structural discrimination was determined from the Support Vector Machine (SVM) technique using five non-truncated (traditional) 3D-PDs. This model showed an external accuracy (i.e., the percentage of correct classifications on the test set) equal to 98.2% (Marrero-Ponce et al., 2020).

Similarly, to the regression models, the classification models were trained (see previous Section) from 16 sets of 3D-PDs corresponding to each truncation function but also contained the traditional non-truncated versions. In summary, 55 3D-PDs (5 non-truncated 3D-PDs and 50 truncated 3D-PDs) conformed each one of the 16 sets. To perform the comparison, models only considering truncated 3D-PDs were built. For each set of 3D-PDs, the Greedy Stepwise search method was coupled with the SVM learner to retain relevant 3D-PDs for the SCOP structural discrimination. This wrapper is implemented in the WEKA software (version 3.8.2) (Witten and Frank, 2005). All the models created were assessed by their accuracies on the test set (Baldi et al., 2000).

**FIGURE 7:**
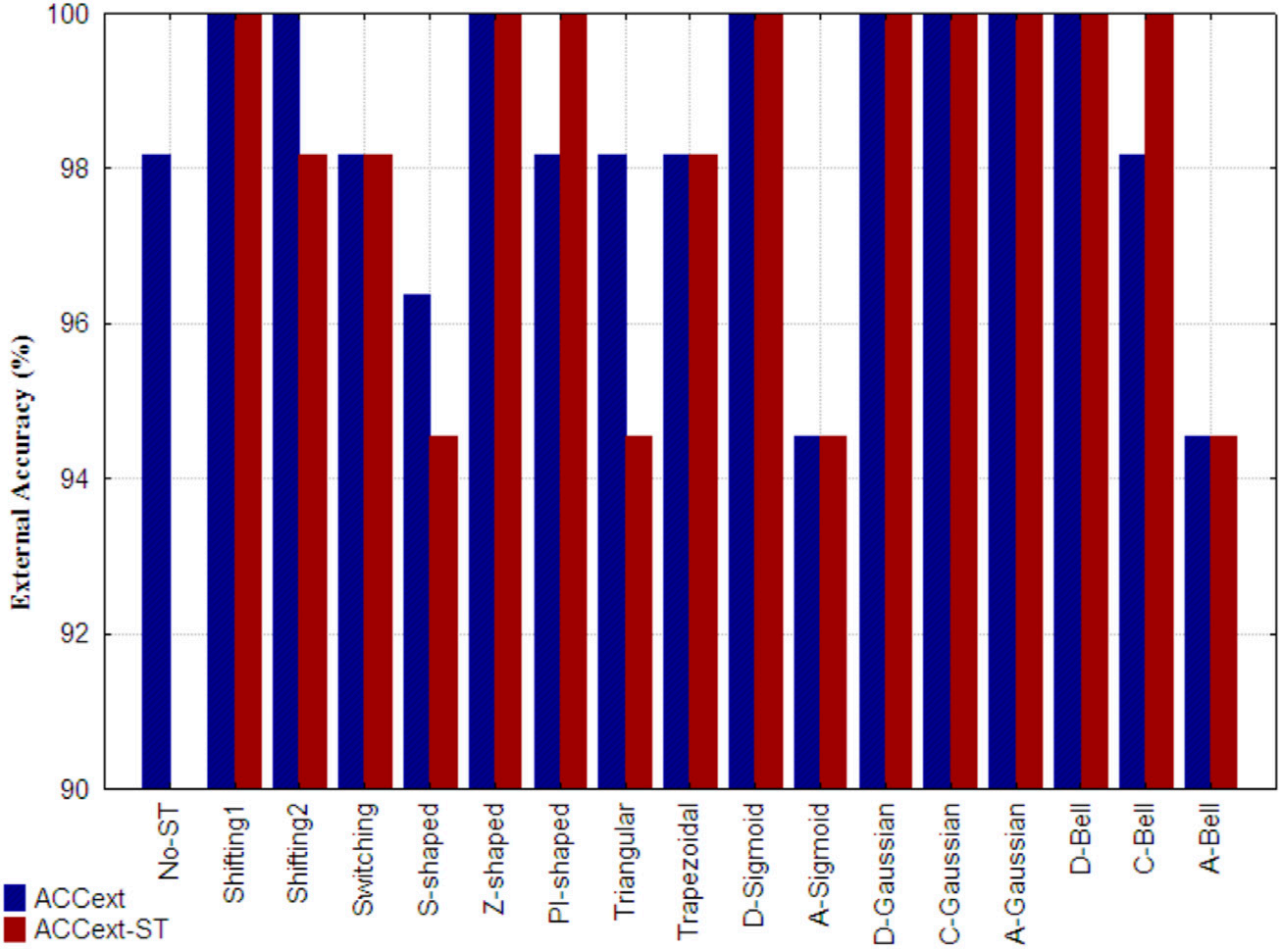
External accuracy attained, by the reference model developed only from classical 3D-PDs (indicated as No-ST), the best model built from each set of truncated 3D-PDs along with the classical 3D-PDs for the prediction of SCOP structural classes (ACC_ext_), as well as the one attained by the best models developed only from truncated 3D-PDs (ACC_ext_-ST). Each set of truncated 3D-PDs employs a traditional function or a fuzzy membership function. All the models were developed with the SVM technique.

Figure 7 shows the external accuracy obtained by the reference model (indicated as No-ST), the accuracies achieved by the models built with non-truncated and truncated 3D-PDs (ACC_ext_), as well as the ones yielded by the models only built with only truncated 3D-PDs (ACC_ext-ST_). It is important to mention that the described wrapper did not retain any non-truncated 3D-PD in the models corresponding to the Switching, Z-shaped, Trapezoidal, D-Gaussian, D-Bell, C-Bell and A-Bell functions. The best results (ACC_ext_) correspond to the models that include 3D-PDs truncated with the Shifting1, Shifting2, Z-shaped, D-Sigmoid, D-Gaussian, C-Gaussian, A-Gaussian and D-Bell functions, because they attained the maximum accuracy (ACC_ext_ = 100%), surpassing the one attained by the reference model. Notably, five out of these six models built with 3D-PDs based on FMFs employ a lower number of 3D-PDs than the used ones by the reference model. Such model complexity reduction by using truncated 3D-PDs based on FMFs suggests that they encode relevant information. However, the models trained with 3D-PDs truncated with TFs were supported with more 3D-PDs than the reference model. Among the best truncation functions mentioned above, the A-Gaussian and C-Gaussian FMFs tend to assign the largest membership values to *aa*s far from the center and in the middle region of the protein, respectively. These findings reinforce the utility of these functions in the truncation of inter-amino acid interactions.

Moreover, the best performance of the models only built with truncated 3D-PDs (ACC_ext-ST_ = 100%) was achieved using 3D-PDs based on the Shifting1, Z-shaped, PI-shaped, D-Sigmoid, D-Gaussian, C-Gaussian, A-Gaussian, C-Bell and D-Bell functions. The other models based showed stable accuracies. In general, the best models including 3D-PDs truncated with FMFs show robust predictions, even considering the non-truncated 3D-PDs.

Additionally, non-truncated 3D-PDs and the truncated derived from the best smoothing functions (i.e., Shifting1, Shifting2, Z-shaped, PI-shaped, D-Sigmoid, D-Gaussian, C-Gaussian, A-Gaussian, D-Bell and C-Bell) were combined into a single dataset, in order to build new models but now considering different types of truncated 3D-PDs. The best model developed carried four 3D-PDs, that showed good performances in both 10-fold cross-validation (ACC_10cv_ = 99.3%) and test dataset (ACC_ext_ = 100%) (see ID: M10, Table 5). The corresponding training/test MCC values were higher than 0.99, suggesting absence of casual correlations. This performance is better than the one achieved by the reference model, as well as the one achieved by each model based on PDs truncated with a same function. Regarding the 3D-PDs entering in the best model, 3 out of 4 are truncated types, which support the hypothesis that an improved description of the 3D protein structure is achieved by combining non-truncated and truncated 3D-PDs, and consequently more accurate prediction models. The C-Gaussian and Shifting1 functions determined the truncated 3D-PDs used in the model above. The C-Gaussian FMF gives more importance to aas placed at the middle of spherical region, suggesting the relevance of this type of functions for protein structural encoding.

According to the outcomes obtained in the two modeling studies previously described, the best smoothing functions are: Z-shaped, D-Sigmoid, Switching, Shifting2, PI-shaped, Shifting1 and A-Gaussian (see SI-4.1). Among them, there are FMFs that tend to assign the highest membership degrees to *aa*s belonging to the three “spherical regions” [i.e.*,* close (e.g., Z-shaped) and distant (e.g., A-Gaussian) from the geometrical center, and belonging to the middle region (e.g., PI-shaped) of the protein]. These results support the application of FMFs in the truncation of inter-amino acid relationships, at contributing to determine 3D-PDs with better predictive ability. Lastly, it is important to indicate the different *r*_*on*_ *-r*_*off*_ intervals that should be considered in further studies. To this end, the 10-top ranked intervals according to the SE-based frequency analysis for each function were analyzed (see SI-5, SI-6 for xml projects). If two 3D-PDs based on different intervals are linearly dependent, then the best-ranked one is preferred. On this basis, the intervals that should be considered are 0–0.4, 0–0.2, 0.2–1, 0.8-1 and 0.4–0.8 for the Z-shaped FMF; 0.2–0.4, 0–0.2, 0–1, 0.8-1 and 0–0.6 for the D-Sigmoid FMF; 0–1, 0–0.6, 0.2–0.4, 0.4–0.6 and 0–0.4 for the PI-shaped FMF; 0–0.6, 0.2–0.4, 0.2–0.6, 0.8-1and 0–0.8 for the A-Gaussian FMF; 0.2–1, 0.2–0.8, 0.6–1, 0.6–0.8 and 0.8-1 for the Switching TF; and 0–0.3, 0–0.4, 0–0.5, 0–0.6 and 0–0.7 for the Shifting2 TF.

### 6.4 Spherically truncated two-linear 3D-PDs for protein folding rate prediction. Comparison with other methods

The modeling ability of the spherically truncated 3D-PDs was shown by building models based on two-linear 3D-PDs truncated with FMFs and TFs for the prediction of protein folding rate. In order to generate the truncated 3D-MDs, 5 out of the 15 previously designed projects (Marrero-Ponce et al., 2020), that define sets of two-linear non-truncated (traditional) 3D-MDs, were selected as baselines to incorporate the truncation method. Particularly, the best truncation functions according to variability, orthogonality and modeling analyses (i.e., Z-shaped, D-Sigmoid, PI-shaped, A-Gaussian, Switching and Shifing2), were jointly inserted with the intervals suggested, were inserted into these projects. These five projects encompass a space of 4330 MDs. These 3D-MDs were computed for each function to build the models using the MuLiMs-MCoMPAs software (Contreras-Torres et al., 2019).

Given the number of 3D-PDs computed, the following procedure was adopted to reduce the dimensionality: the 3D-MDs computed from each truncation function class were merged into a dataset (one per truncation function, see SI-7); the 2000 top-ranked 3D-PDs were selected from the previous dataset, according to their SE values, by using the IMMAN software (Urias et al., 2015); the 3D-MDs showing pairwise correlation greater than 0.95 were deleted; relevant 3D-MDs were selected by applying the Correlation Feature Selection (CFS) filter implemented on the WEKA package (version 3.8.2) (Hall, 1999). The resulting datasets were merged into a single dataset, which was used to build the regression models by using the GA-MLR wrapper available in the MobyDigs software. The GA was set up as follows: the maximum number of descriptors was 4; the leave-one-out cross-validation method (Q2loo) was used as fitness function; the crossover/mutation rate was spanned from 0 to 1 starting at 0.5; and the selection methods considered were random, roulette and tournament. The MLR models obtained were internally validated through the bootstrapping (Q2boot) (Leger et al., 1992) and Y-scrambling (a(Q2)) (Tropsha, 2010) procedures. Moreover, the external correlation coefficient (Q2ext) was employed to estimate the prediction ability of the models on the test set.

The MLR equation and their statistical parameters corresponding to the best model obtained are indicated in Eq. 8 and Table 3 (ID: M5), respectively. Based on prediction errors on the test set, the following proteins: pdb1a6n, pdb1psf, pdb1ris and pdb1spr_A were considered as outliers (see also SI-8).

$$\begin{array}{l} \ln\left(kf\right)=4.57055\left(\pm 0.8215\right)\times A-0.50604\left(\pm 0.15212\right)\\ \times B-0.05873\left(\pm 0.00649\right)\times C-3.16702\left(\pm 1.839833\right)\times D+14.63633 \end{array}$$ (5)

where, *A* = AB_Q1_F_M32_SS-1_o_T_LGP[+12.0]_LGL[4–5.9]_LGST(AGAUSSIAN)[0–0.8]_KDS; *B* = AB_S_F_M7_MP-1_T_LGP[+12.0]_LGL[4–5.9]_LGST(SWITCHING)[0.6–1]_PBS; *C* = AVG_N1_Q_M24_SS-6_o_T_LGP[1–3]_LGL[8.1–11]_LGST(Z)[0.2–1]_PAH; *D* = AVG_I50_Q_M8_NS-3_o_RPU_LGST(Z)[0.8–1]_PBS

Concerning the training results, it can be noted that this model presents a good result of fitting (R2 = 80.21), correlation (Q2loo = 77.28) and robustness (Q2boot = 76.69). Moreover, the Y-scrambling index (a(Q2)) is less than -0.12, indicating training results were not obtained by chance. On the other hand, the results on the external set (Q2ext) were greater than 95% suggesting the high predictive power of this model, which reached an improvement of 8.3% respect the reference model (see Section 6.2). The analyze statistical parameters evidence that the truncated 3D-MDs encode relevant structural information.

Regarding the PDs entering this model, the presence of the A-Gaussian, the Switching and Z-shaped truncation functions (with a higher occurrence of Z-shaped) called the attention because the combination of 3D-MDs based on the Z-shaped and the Switching functions improved predictions in a previous subsection. Table 4 lists the statistical parameters corresponding to the proposed model (denoted as M5) and to the previously reported ones. This table shows that the correlation coefficient attained by the best model built surpasses those from the existing approaches, which confirm that the truncation methodology proposed constitutes a promising tool to encode relevant and singular structural features.

**TABLE 4:** Performances of several existing models versus the presented approach, in the prediction of protein folding rates.

Descriptors	Descriptor dimension	Q^2^ (%) (*training*)	SDEP (*training*)	Q^2^ (%) (*test*)	SDEP *(test)*
Previous Studies					
Folding degree (Ruiz-Blanco et al., 2015)	3D	73.96	2.20	54.76	2.03
Long Range Order (Gromiha and Selvaraj, 2001)	3D	72.25	2.28	—	—
Contact order (Plaxco et al., 1998)	3D	73.96	2.19	—	—
Total Contact Distance (Zhou et al., 2002)	3D	73.96	2.21	—	—
FoldRate web server (Chou and Shen, 2009)	1D	77.44	2.03	—	—
Generalized two-linear PDs (Marrero-Ponce et al., 2020)	3D	75.30	2.15	87.50	2.05
Generalized three-linear PDs (Terán et al., 2019)	3D	74.80	2.17	85.75	2.78
This study					
Two-linear MDs truncated with FMFs and TFs (M5)	3D	77.28	2.06	95.82	0.61

### 6.5 Alignment-free prediction of SCOP structural classes using spherically truncated 3D-MDs. Comparison with other methods

The modeling ability of the spherically truncated 3D-MDs is illustrated by building alignment-free models based on 2-linear 3D-MDs truncated with FMFs and TFs for the prediction of the SCOP structural classes. In order to calculate the truncated 3D-MDs, the same 5 out of 15 previously designed projects (Marrero-Ponce et al., 2020) to compute two-linear non-truncated 3D-MDs were selected as baselines to introduce the truncation method. In particular, the best truncation functions, according to variability, orthogonality and modeling analyses, together with their corresponding best intervals were incorporated into these projects. The total number of 3D-MDs defined from these projects is equal to 4330, which were computed for each truncation function using the MuLiMs-MCoMPAs software (Contreras-Torres et al., 2019). Given the high number of 3D-MDs, the following strategy was performed for each function: the 3D-MDs computed from each project were merged into a single dataset; the best-ranked 1000 3D-MDs, according to SE values, were selected from the aforementioned datasets; relevant 3D-MDs were selected through the Correlation Feature Selection (CFS) (Hall, 1999) filter implemented in the WEKA package (Witten and Frank, 2005); and the resulting datasets were fused into a single one that was subsequently used to determine the prediction models.

Subsequently, the Greedy Stepwise search method was jointly used with the SVM and Linear Discriminant Analysis (LDA) methods, respectively, in order to retain relevant 3D-MDs for the structural class classification. The Pearson universal kernel “PUK” was used in the SVM model. This wrapper was applied through the WEKA software (Witten and Frank, 2005). Table 5 lists the statistical parameters of the models developed, as well as the best model only built with the non-truncated 3D-MDs (model M10). Here, the created SVM (M10 and M11) and LDA (M12)-based models achieved accuracies of 99.3% and 100% on cross-validation and external set tests. Thus, they outperformed the classification measures attained by the reference model. Of the three models, the LDA model is the least complex at employing only three 3D-MDs.

**TABLE 5:** Best models built up for the structural discrimination of 204 proteins using truncated and classical MuLiMs-MCoMPAs 2-linear PDs.

Model ID	Method	Number of MDs (based on FMFs-TFs)	ACC (%) training (149)*	MCC training	ACC (%) test (55)	MCC test
M10	SVM	4(2–1)	99.33	0.99	100	1
M11	SVM	6(5–1)	99.33	0.99	100	1
M12	LDA	3(2–1)	99.33	0.99	100	1

*Results obtained from a 10-fold cross-validation test.

Regarding the truncated 3D-MDs included in the SVM model, it is important to remark that these were obtained with the Switching, Z-shaped, D-Sigmoid and A-Gaussian truncation functions; whereas the truncated 3D-MDs included in the LDA model were calculated with the Switching, Z-shaped and A-Gaussian truncation functions. The recurrence of Switching-Z-shaped-A-Gaussian triplet, similarly in the MLR model development shown in the previous subsection, as well as the reappearance of Switching-Z-shaped pair, suggest these functions provide complementary MDs in terms of structural space for modeling purposes (see also SI-8).

A comparison between the best model developed regarding several methods reported in the literature to predict protein structural classes is shown in Table 6. From this Table, it can be observed that the LDA model performance was superior to the ones attained by several existing models. All in all, it can be stated that the spherical truncation method based on FMFs is a valuable tool to increase the structural information codified by non-truncated 3D-MDs.

**TABLE 6:** Comparison of existing SCOP structural predictors versus this approach.

Protein Descriptors	Accuracy (%) Training	Accuracy (%) Test
Previous Studies		
AA composition (Cai et al., 2006)	83.80	—
Pseudo AA composition (Zhang et al., 2008)	91.20	—
Pair coupled AA composition (Cai et al., 2002)	74.50	—
PSI-BLAST (Chen et al., 2008)	94.10	—
Bilinear descriptors (Marrero Ponce et al., 2015)	92.60	92.70
Generalized two-linear 3D-PDs (Marrero-Ponce et al., 2020)	98	98.20
Generalized two-linear and three-linear 3D-PDs (Terán et al., 2019)	99.33	98.20
This study		
Two-linear 3D-MDs truncated with FMFs (SVM) (M10)	99.33	100
Two-linear 3D-MDs truncated with FMFs and TFs (LDA) (M12)	99.33	100

## 7 Conclusion

The introduction of fuzzy spherical truncation functions for the definition of 3D multi-linear PDs has shown to encode orthogonal information to the one obtained from the traditional (non-truncated) 3D-PDs. These functions also enhanced the information discrimination capacity of the MuLiMs-MCoMPAs 3D-PDs.

The evaluation of sixteen “membership functions” allowed to find a set of six functions that ranked the highest in terms of the exploratory, variability, orthogonality and modeling analyses: Z-shaped, Shitting1, D-Sigmoid, PI-shaped, Switching and A-Gaussian. These selected functions favor the inter amino acids’ interactions near to center, the middle and outer regions from the protein’s geometrical center.

The modeling studies carried out illustrated the usefulness of the truncated 3D-PDs. These novel indices yielded more robust and accurate predictions than the ones achieved by several existing models. Particularly, combinations of 3D-PDs based on the A-Gaussian, Switching and Z-shaped truncation functions show high modeling power in the prediction of protein folding rate and SCOP structural classes.

Based on variability, collinearity, and prediction capability analyses of every theoretical component of the indices, 26 and 14 projects were generated for the two-linear and three-linear indices, respectively. The complete configuration for the two-and three-linear indices is shown in Table SI1-1, SI1-2, respectively. These 40 projects allow users to retrieve more informative 3D-PDs, save time at performing separate analyses and to focus on evaluating the intended protein function or property.

Future study includes the development of a version of the MuLiMs-MCoMPAs module to compute truncated 3D-PDs by using some extensions of fuzzy sets that employ three-dimensional membership functions (like SFS) with a more detailed description, informatively and explicitly (Kutlu Gündoğdu and Kahraman, 2019; Donyatalab et al., 2020), which could give better 3D-PDs.

## Data Availability

The original contributions presented in the study are publicly available. This data can be found here: https://doi.org/10.6084/m9.figshare.19947788.v1.

## Abbreviations

3D-MD, Geometrical Molecular Descriptor; 3D-PD, Geometrical Protein Descriptor; QSAR, Quantitative Structure-Activity Relationships; QuBiLS-MIDAS, Quadratic, Bilinear & N-Linear Maps based on N-Metric Matrices and Atomic Weightings; MuLiMs-MCoMPAs, Multi-Linear Maps based on N-Metric & Generalized Contact Matrices and Amino Acid Weightings; SE, Shannon’s Entropy; TF, Traditional Function; FMF, Fuzzy Membership Function.
